# Supplementary material for: Association of thyroid hormones with the severity of chronic kidney disease: a cross-sectional observational study at Tabuk, Saudi Arabia
Source: PeerJ. 2024 Oct 28;12:e18338. doi: 10.7717/peerj.18338 (PMC11526795; doi:10.7717/peerj.18338)
Supplement: Supplemental Information 2 [file peerj-12-18338-s002.docx]

**Supplementary Table 1. Baseline Characteristics Of The Patients**

| **MRN** | **S.No.** | **Sex** | **Age** | **Weight** | **BP** | **TSH** | **FT4** | **FT3** | **PTH** | **BUN** | **Creatinine** | **Na** | **K** |
| --- | --- | --- | --- | --- | --- | --- | --- | --- | --- | --- | --- | --- | --- |
| 394333 | 1 | F | 59 | 70 | **142/78** | 0.8 | 15 | 5.17 | 33.68 | 5.9 | 247 | 131 | 4.5 |
| 199213 | 2 | M | 52 | 85 | **130/90** | 0.43 | 21 | 5.31 | 34.98 | 5.60 | 253.00 | 138.00 | 3.52 |
| 394251 | 3 | F | 57 | 100 | **132/76** | 3.1 | 14 | 4.43 | 72.42 | 20.90 | 514.00 | 138.00 | 4.94 |
| 412441 | 4 | M | 54 | 70 | **150/80** | 0.51 | 20 | 4.72 | 13.86 | 17 | 391.00 | 140.00 | 4.92 |
| 31032 | 5 | M | 31 | 60 | **151/69** | 3.60 | 21 | 6.4 | 66.54 | 22.3 | 771 | 137 | 6.18 |
| 10851 | 6 | M | 59 | 117 | **182/101** | 4.1 | 22 | 5.13 | 109.9 | 32 | 950 | 133 | 5.75 |
| 306250 | 7 | F | 70 | 80 | 149/77 | 2.75 | 19 | 5.9 | 20.21 | 20.00 | 292 | 141 | 4.25 |
| 325559 | 8 | M | 43 | 88 | **140/95** | 3.48 | 18 | 3.88 | 273.4 | 22.5 | 1326 | 137.00 | 5.03 |
| 150714 | 9 | M | 20 | 49 | **163/117** | 0.42 | 16 | 5.29 | 47.57 | 11.9 | 729.00 | 138.00 | 6.39 |
| 73488 | 10 | M | 57 | 70 | **118/79** | 1.37 | 17 | 4.86 | 16.82 | 8.9 | 348 | 134.00 | 5.12 |
| 220522 | 11 | M | 74 | 70 | **166/82** | 3.22 | 20 | 4.13 | 32.3 | 28 | 173 | 135.00 | 5.45 |
| 64273 | 12 | M | 65 | 80 | **148/80** | 0.90 | 12 | 5.3 | 61.9 | 7.3 | 357 | 130.00 | 4.25 |
| 381944 | 13 | F | 69 | 65 | **180/75** | 0.62 | 21 | 6.8 | 39.47 | 17.8 | 601 | 135.00 | 4.11 |
| 81113 | 14 | M | 58 | 110 | **152/82** | 1.20 | 18 | 3.5 | 37.41 | 12.3 | 450 | 134.00 | 4.28 |
| 363870 | 15 | F | 72 | 68 | **116/53** | 2.54 | 21 | 4.55 | 17.81 | 17.50 | 295.00 | 138.00 | 4.83 |
| 413074 | 16 | M | 21 | 80 | **150/90** | 3.36 | 19 | 3.43 | 73.36 | 7.50 | 336.00 | 140.00 | 4.51 |
| 413505 | 17 | F | 73 | 75 | **128/70** | 1.12 | 17 | 3.6 | 47.89 | 18.2 | 223 | 132 | 4.62 |
| 413864 | 18 | M | 60 | 80 | 150/65 | 2.5 | 20 | 5.67 | 42.2 | 8.5 | 316 | 139 | 4.39 |
| 257563 | 19 | F | 49 | 95 | **147/51** | 3.16 | 22 | 3.83 | 16.8 | 14 | 115 | 126 | 4.78 |
| 39720 | 20 | M | 52 | 70 | **181/88** | 1.79 | 16 | 4.30 | 59.3 | 20.2 | 136 | 128 | 5.44 |
| 205164 | 21 | M | 82 | 80 | **91/44** | 3.22 | 19 | 3.17 | 38.18 | 11.6 | 452 | 137 | 4.22 |
| 350462 | 22 | F | 63 | 90 | **153/62** | 2.7 | 21 | 5.75 | 17.40 | 7.5 | 320.3 | 137 | 4.3 |
| 339047 n | 23 | F | 30 | 60 | 166/81 | 2.0 | 20 | 4.23 | 61.3 | 4.9 | 70 | 138 | 4.25 |
| 202262 | 24 | F | 69 | 60 | **100/68** | 1.4 | 13 | 6.6 | 23.8 | 16.6 | 364 | 130 | 5.08 |
| 41717 | 25 | F | 48 | 80 | **130/66** | 2.8 | 17 | 3.28 | 63.7 | 12.2 | 299 | 137 | 4.27 |
| 16641 | 26 | F | 77 | 77 | **200/84** | 2.96 | 20 | 5.9 | 88.23 | 12.9 | 489.2 | 139 | 5.05.02 |
| 288340 | 27 | M | 59 | 70 | **184/72** | 3.21 | 16 | 3.12 | 42.5 | 14.1 | 259 | 140 | 2.95 |
| 123127 | 28 | M | 61 | 67 | **184/72** | 1.88 | 14 | 3.5 | 33.2 | 7.2 | 410 | 143143 | 4.29 |
| 11582 | 29 | F | 66 | 63 | **164/80** | 2.9 | 17 | 6.28 | 20.9 | 12.9 | 221 | 130 | 5.02 |
| 93044 | 30 | M | 44 | 66 | **190/89** | 2.50 | 22 | 3.7 | 16.7 | 13.1 | 312 | 127 | 4.54 |
| 401531 | 31 | M | 30 | 70 | 108/62 | 1.93 | 19 | 6.3 | 99.5 | 15.5 | 781.5 | 132 | 3.8 |
| 25215 | 32 | F | 76 | 70 | 190/83 | 1.40 | 20 | 3.3 | 57.77 | 19.2 | 232 | 141 | 4.96 |
| 29850 | 33 | M | 65 | 75 | 180/86 | 3.52 | 18 | 3.68 | 76.9 | 15.5 | 228 | 140 | 3.75 |
| 17158 | 34 | F | 56 | 76 | 160/65 | 1.74 | 22 | 6.5 | 17.7 | 8.6 | 254 | 137 | 3.94 |
| 375960 | 35 | F | 61 | 70 | 91/40 | 2.78 | 22 | 4 | 47.5 | 15.5 | 449 | 136 | 4.52 |
| 327363 | 36 | M | 57 | 72 | **177/76** | 3.65 | 14 | 6.7 | 10.9 | 8.5 | 278 | 134 | 4.81 |
| 321563 | 37 | M | 23 | 102 | 130/75 | 2.18 | 18 | 3.9 | 49.3 | 24.2 | 349 | 136 | 4.65 |
| n-337409 | 38 | F | 76 | 41 | **121/81** | 2.43 | 20 | 5.22 | 10.7 | 12.1 | 145 | 139 | 4.49 |
| 399518 | 39 | M | 65 | 80 | 182/70 | 3.49 | 13 | 6.13 | 37.01 | 12.3 | 352.00 | 146 | 5.9 |
| 272491 | 40 | M | 34 | 87 | **145/84** | 3.09 | 19 | 5.9 | 21.26 | 11.40 | 293.00 | 143.00 | 5.08 |
| 109082 | 41 | F | 52 | 70 | **175/86** | 2.29 | 16 | 6.24 | 28.4 | 8.4 | 137 | 138.00 | 4.72 |
| 102455 | 42 | F | 45 | 70 | **122/58** | 3.27 | 12 | 5.23 | 22.7 | 14 | 329.00 | 142 | 4.81 |
| 20159 | 43 | F | 82 | 75 | **152/78** | 3.38 | 12 | 3.90 | 14.59 | 18.80 | 305.00 | 133.00 | 5.16 |
| 264208 | 44 | F | 26 | 65.6 | **104/55** | 3.22 | 14 | 4.3 | 68.4 | 24 | 536.00 | 153 | 3.9 |
| 203622 | 45 | M | 56 | 96 | **109/77** | 2.93 | 20 | 3.87 | 12.0 | 3.7 | 158 | 136 | 3.74 |
| 341499 | 46 | M | 59 | 80 | 130/63 | 2.10 | 14 | 6.73 | 24.6 | 20.3 | 427 | 137 | 4.7 |
| 222810 | 47 | M | 57 | 70 | 105/70 | 3.81 | 17 | 5.88 | 10.77 | 13 | 220 | 141 | 4.6 |
| 781 | 48 | F | 56 | 60 | 133/65 | 4.21 | 16 | 4.7 | 18.02 | 15.6 | 246.00 | 144 | 4.43 |
| 212890 | 49 | M | 55 | 70 | 129/77 | 2.49 | 22 | 6.29 | 24.11 | 8.6 | 171.00 | 142 | 4.76 |
| 274941 | 50 | F | 51 | 75 | 140/53 | 2.52 | 12 | 3.9 | 11.28 | 9.5 | 138.00 | 143 | 3.98 |
| 131986 | 51 | F | 64 | 80 | 122/62 | 1.27 | 15 | 3.68 | 39.6 | 9.5 | 159.00 | 144 | 4.25 |
| 73722 | 52 | F | 77 | 80 | **170/87** | 3,71 | 21 | 3.98 | 12.83 | 10.6 | 154.00 | 131 | 4.64 |
| 362683 | 53 | M | 37 | 80 | 128/70 | 3.80 | 13 | 4.50 | 10.56 | 7.6 | 213.00 | 141 | 4.86 |
| 16152 | 54 | F | 66 | 95 | **118/57** | 3.0 | 22 | 5.16 | 13.55 | 12.9 | 144 | 140 | 4.9 |
| 338282 | 55 | F | 22 | 60 | 132/76 | 2.9 | 17 | 3.95 | 22.7 | 13.9 | 103 | 146 | 4.56 |
| 480 | 56 | F | 73 | 88 | 144/82 | 3.10 | 20 | 4.81 | 29.2 | 8.4 | 115 | 138 | 5.07 |
| 249567 | 57 | M | 57 | 84 | **154/70** | 2.11 | 16 | 4.88 | 17.5 | 8.8 | 133 | 140 | 4.7 |
| 2756 | 58 | F | 71 | 70 | 105/54 | 4.07 | 18 | 5.0 | 64.4 | 10.6 | 117 | 139 | 4.58 |
| 25913 | 59 | M | 71 | 75 | 134/82 | 3.22 | 20 | 4.25 | 17.2 | 9.9 | 172 | 141 | 5.10 |
| 205164 | 60 | M | 82 | 80 | **91/44** | 3.15 | 20 | 3.10 | 49.17 | 18.9 | 534 | 145 | 4.55 |
| 184855 | 61 | M | 87 | 75 | 119/70 | 4.2 | 17 | 6.73 | 39.2 | 19.2 | 655 | 140 | 4.52 |
| 313241 | 62 | M | 57 | 80 | 121/65 | 0.62 | 21 | 3.0 | 37.6 | 16.8 | 223 | 137 | 3.66 |
| 116793 | 63 | M | 55 | 103 | 169/88 | 4.17 | 20 | 6.4 | 16.1 | 9.2 | 157 | 139 | 4.09 |
| 422382 | 64 | M | 22 | 70 | **140/100** | 2.13 | 19 | 4.49 | 12.67 | 8.3 | 226.00 | 135 | 4.6 |
| 165573 | 65 | F | 59 | 100 | 125/72 | 3.2 | 16 | 4.38 | 23.29 | 32.4 | 253 | 148 | 4.49 |
| 29105 | 66 | M | 91 | 70 | 133/60 | 0.66 | 22 | 3.24 | 11.2 | 8.4 | 157 | 138 | 4.9 |
| 72848 | 67 | M | 69 | 63.6 | **161/66** | 2.34 | 17 | 5.30 | 12.75 | 7.60 | 145.00 | 138.00 | 4.12 |
| 15626 | 68 | F | 56 | 81 | **154/83** | 3.11 | 13 | 6.0 | 39.55 | 21.10 | 278.00 | 138 | 4.93 |
| 110877 | 69 | M | 82 | 90 | 120/73 | 0.94 | 19 | 5.5 | 19.84 | 15.10 | 249.00 | 145 | 5.49 |
| 23927 | 70 | F | 60 | 60 | 110/78 | 2.51 | 15 | 5.39 | 26.13 | 18.9 | 345 | 138 | 2.87 |
| 69541 | 71 | F | 59 | 80 | **151/91** | 3.7 | 21 | 4.7 | 13.25 | 18.60 | 375.00 | 138 | 4.68 |
| 209747 | 72 | F | 78 | 50 | 110/66 | 1.5 | 21 | 3.89 | 117.6 | 19.20 | 496.00 | 137.00 | 4.58 |
| 152992 | 73 | F | 70 | 90 | 150/85 | 3.4 | 16 | 4.42 | 21.79 | 19.90 | 267.00 | 137 | 4.8 |
| 89019 | 74 | M | 63 | 75 | 129/75 | 0.92 | 18 | 5.22 | 59.8 | 9.4 | 359 | 134 | 5.0 |
| 397070 | 75 | M | 69 | 60 | 140\70 | 4.2 | 22 | 5.8 | 11.82 | 20.30 | 195.2 | 135.00 | 4.21 |
| 400106 | 76 | M | 26 | 75 | 141\60 | 1.77 | 21 | 4.81 | 9.3 | 18.9 | 385.5 | 136 | 4.2 |
| 278295 | 77 | F | 63 | 64.4 | 172\75 | 0.42 | 16 | 5.33 | 9.3 | 12.3 | 451 | 137 | 3.73 |
| 415281 | 78 | F | 58 | 70 | 138\64 | 2.9 | 20 | 5.16 | 9.5 | 8.8 | 114.00 | 139 | 3.9 |
| 323013 | 79 | M | 78 | 86 | 121\65 | 0.74 | 22 | 6.49 | 8.8 | 14.7 | 256 | 138 | 4.88 |
| 338883 | 80 | F | 86 | 70 | 130\80 | 2.37 | 13 | 6.62 | 9.06 | 34.3 | 313 | 141 | 5.6 |
| 305019 | 81 | M | 56 | 80 | 176\68 | 1.18 | 15 | 4.90 | 9.7 | 7 | 386 | 137 | 4.96 |
| 126002 | 82 | F | 23 | 30 | 154\59 | 2.93 | 18 | 3.6 | 8.6 | 10.2 | 136 | 138 | 4.7 |
| 222810 | 83 | M | 57 | 70 | 143\92 | 2.73 | 21 | 6.2 | 10.77 | 13 | 220 | 141 | 4.6 |
| 220003 | 84 | F | 83 | 80 | 136\71 | 2.93 | 19 | 3.17 | 9.25 | 11.10 | 135.00 | 138 | 3.87 |
| 22561 | 85 | F | 78 | 94 | 158\73 | 1.43 | 14 | 6.4 | 8.86 | 13 | 133 | 138 | 4.6 |
| 27832 | 86 | M | 77 | 79 | 112/56 | 2.33 | 17 | 4.12 | 11.38 | 9.7 | 152 | 142 | 3.74 |

| **Parameter** | **N.R** | **min** | **max** | **unit** |
| --- | --- | --- | --- | --- |
| TSH |  | **0.27** | **4.2** | **µiU/ml** |
| FT3 |  | **3.1** | **6.8** | **pmol/L** |
| FT4 |  | **12** | **22** | **pmol/L** |
| PTH |  | **1.59** | **6.89** | **pmol/L** |
| NA |  | **135** | **152** | **mmoll/L** |
| K |  | **3.5** | **5.1** | **MMOL/L** |
| BUN |  | **3.2** | **8.2** | **uMOL/L** |
| Creatinine |  | **62** | **106** | **µmol/L** |
