# Supplementary material for: Association of thyroid hormones with the severity of chronic kidney disease: a cross-sectional observational study at Tabuk, Saudi Arabia
Source: PeerJ. 2024 Oct 28;12:e18338. doi: 10.7717/peerj.18338 (PMC11526795; doi:10.7717/peerj.18338)
Supplement: Supplemental Information 3 [file peerj-12-18338-s003.docx]

**STROBE Statement—checklist of items that should be included in reports of observational studies**

|  | **Item No.** | **Recommendation** | **Page No.** | **Relevant text from ttmanuscript** |
| --- | --- | --- | --- | --- |
| **Title and abstract** | 1 | (*a*) Indicate the study’s design with a commonly used term in the title or the abstract | 2 | Cross-Sectional Observational Study |
|  |  | (*b*) Provide in the abstract an informative and balanced summary of what was done and what was found | 2 | Background: The interaction between chronic kidney disease (CKD) and thyroid dysfunction is increasingly recognized in the medical field. Further exploration is needed to fully comprehend the implications of this relationship.  Objective: This study seeks to assess the link between thyroid hormone levels (including TSH, T3, and T4) and renal disease severity markers (such as creatinine, urea, and PTH) in CKD patients.  Methods: A cross-sectional observational study involved 86 CKD participants from King Fahad Hospital in Tabuk. Biochemical parameters were measured using a Roche Cobas E411 analyzer, and the Pearson correlation coefficient was used to analyze the associations.  Results: The analysis revealed a weak correlation between thyroid hormones and renal disease markers, with no significant correlation between creatinine/TSH, T3, T4, or between urea and thyroid hormones. However, a strong positive correlation was found between PTH and creatinine.  Conclusion: Thyroid hormone levels show minimal correlation with renal disease severity, while the significant link between PTH and creatinine emphasizes its importance in CKD management. Further research is needed to explore potential therapeutic avenues for thyroid dysfunction in CKD patients. |
| **Introduction** |  |  |  |  |
| Background/rationale | 2 | Explain the scientific background and rationale for the investigation being reported | 3-4 | The study aimed to evaluate the correlation between thyroid hormone levels and renal disease severity indicators in individuals with CKD, highlighting the importance of monitoring thyroid function in renal disease patients.  The investigation utilized a cohort of 86 participants from a renal clinic, analyzing thyroid hormone concentrations and renal function markers to understand potential therapeutic targets for thyroid dysfunction in CKD patients.  The study employed the Roche Cobas E411 analyzer for quantitative assessment of biochemical parameters and thyroid hormone levels, emphasizing the need for further research to explore pathophysiological relationships and therapeutic interventions in this population. |
| Objectives | 3 | State specific objectives, including any prespecified hypotheses | 4 | The research aimed to assess the relationship between thyroid hormone levels and markers of renal disease severity in individuals with CKD.  The investigation aimed to identify potential therapeutic avenues for treating thyroid dysfunction in CKD patients.  A cohort of 86 participants from a renal clinic was utilized to examine thyroid hormone levels and renal function markers.  The study aimed to reveal connections between thyroid hormone levels and kidney pathology indices to devise targeted interventions for enhanced patient outcomes. Regression analyses were used to determine the predictive capability of thyroid indicators concerning renal function markers. |
| **Methods** |  |  |  |  |
| Study design | 4 | Present key elements of study design early in the paper | 4-5 | The cross-sectional observational study included 86 participants with CKD, assessing biochemical parameters and thyroid hormone concentrations with a Roche Cobas E411 analyzer.  The Pearson correlation coefficient was utilized to analyze the associations between thyroid function markers and renal disease indicators.  Regression analyses were carried out to assess the predictive power of thyroid indicators regarding renal function markers.  The study aimed to elucidate the associations between thyroid hormones, renal markers, and parathyroid hormone in individuals with CKD. |
| Setting | 5 | Describe the setting, locations, and relevant dates, including periods of recruitment, exposure, follow-up, and data collection | 4-5 | The research was carried out at the renal clinic of King Fahad Hospital in Tabuk, involving 86 participants with CKD.  Data collection and participant recruitment took place within the specified timeframe at the hospital.  Biochemical parameters and thyroid hormone concentrations were evaluated using a Roche Cobas E411 analyzer following the manufacturer's guidelines. Regression analyses were employed to assess the predictive strength of thyroid indicators regarding renal function markers. The dataset was analyzed using SPSS software, with correlation and linear regression analyses conducted to examine the relationship between thyroid parameters and kidney function metrics. |
| Participants | 6 | (*a*) *Cohort study*—Give the eligibility criteria, and the sources and methods of selection of participants. Describe methods of follow-up  *Case-control study*—Give the eligibility criteria, and the sources and methods of case ascertainment and control selection. Give the rationale for the choice of cases and controls *Cross-sectional study*—Give the eligibility criteria, and the sources and methods of selection of  participants | 4-6 | **Eligibility Criteria and Participant Selection Methods in Different Study Designs:**  **Cohort Study:**  Inclusion Criteria: Adult candidates with renal pathologies indicated by elevated serum creatinine and blood urea levels.  Exclusion Criteria: Excluded healthy individuals, dialysis patients, and those with concurrent systemic diseases  **Case-Control Study:**  Eligibility Criteria: Cases and controls selected based on specific criteria related to the research question.  **Case Ascertainment:** Cases are identified from individuals with the condition of interest, while controls are selected without the condition.  **Control Selection:** Controls chosen to be similar to cases in certain characteristics, such as age, gender, or other relevant factors.  Cross-Sectional Study:  **Eligibility Criteria:** Participants are selected based on specific characteristics or conditions being studied.  **Selection Methods:** Participants were recruited from a specific population or setting for a one-time assessment of variables of interest. |
|  |  | (*b*) *Cohort study*—For matched studies, give matching criteria and number of exposed and unexposed  *Case-control study*—For matched studies, give matching criteria and the number of controls per  case |  |  |
| Variables | 7 | Clearly define all outcomes, exposures, predictors, potential confounders, and effect modifiers.  Give diagnostic criteria, if applicable | 4-6 | Outcomes: Severity of renal disease markers, including serum creatinine, urea, and parathyroid hormone levels  Exposures: Thyroid hormone levels, including thyroid-stimulating hormone (TSH), triiodothyronine (T3), and thyroxine (T4)  Predictors: Thyroid indicators concerning renal function markers, such as TSH, T3, and T4 |
| Data sources/ measurement | 8* | For each variable of interest, give sources of data and details of methods of assessment (measurement). Describe comparability of assessment methods if there is more than one group | 4-6 | Thyroid Hormone Levels: Data obtained from biochemical analysis using a Roche Cobas E411 analyzer.  Renal Disease Markers: Assessed through biochemical scrutiny of blood specimens.  Measurement Techniques: Assays for T3, PTH, creatinine, TSH, and urea were conducted using the Roche Cobas E411 analytical platform  Comparability of Assessment Methods: Standardized measurement techniques were employed across all groups, ensuring consistency in data collection and analysis. |
| Bias | 9 | Describe any efforts to address potential sources of bias | 4-6 | Utilization of standardized measurement techniques to ensure consistency in data collection and analysis.  Conducting regression analyses to ascertain statistically significant associations and potential functional dependencies between thyroid hormone levels and renal markers. |
| Study size | 10 | Explain how the study size was arrived at | 4-6 | Sample size estimation was executed utilizing the Raosoft calculator, considering the target population size, hypothesized prevalence, margin of error, and desired confidence level  Raosoft computed a minimum sample size of 80 to achieve a 95% confidence level, adjusted to 86 to align with similar precedent studies |
| Continued on next page |  |  |  |  |

| Quantitative variables | 11 | Explain how quantitative variables were handled in the analyses. If applicable, describe which groupings were chosen and why. **Page** **4-6**  ***Quantitative variables, including thyroid hormone levels and renal function markers, were assessed using correlation and linear regression analyses to evaluate statistical dependence and potential predictive relationships***  ***The dataset was analyzed using SPSS software, with Pearson correlation coefficients computed to evaluate the statistical dependence between thyroid endocrine parameters (TSH, FT4, FT3) and kidney function metrics (serum creatinine, blood urea nitrogen, K, Na)***  ***Regression analyses were employed to ascertain the predictive potency of thyroid indicators concerning renal function markers, aiming to discern statistically significant associations and potential functional dependencies*** |
| --- | --- | --- |
| Statistical methods | 12 | 1. Describe all statistical methods, including those used to control for confounding **Page 4-6**   ***Pearson correlation coefficients were computed to evaluate the statistical dependence between thyroid hormones and renal disease markers***  ***Regression analyses were utilized to ascertain the predictive potency of thyroid indicators concerning renal function markers***  ***The study employed statistical techniques to control for confounding variables and assess the relationships between thyroid hormones and renal function markers*** |
|  |  | 1. Describe any methods used to examine subgroups and interactions   ***Subgroup analyses were not explicitly mentioned in the provided sources***  ***The study primarily focused on evaluating the correlation between thyroid hormone levels and renal disease severity in individuals with chronic kidney disease***  ***Further research with a larger sample size and longitudinal evaluation was suggested to confirm findings and address limitations***  ***The study utilized regression analyses to discern statistically significant associations and potential functional dependencies between thyroid hormone levels and indices of kidney pathology*** |
|  |  | (*c*) Explain how missing data were addressed *NA* |
|  |  | (*d*) *Cohort study*—If applicable, explain how loss to follow-up was addressed *NA*  *Case-control study*—If applicable, explain how the matching of cases and controls was addressed *Cross-sectional study*—If applicable, describe analytical methods taking account of the sampling strategy *NA* |
|  |  | (*e*) Describe any sensitivity analyses: ***The study did not mention conducting sensitivity analyses to assess the robustness of the results*** |
| **Results** |  |  |
| Participants | 13* | 1. Report numbers of individuals at each stage of study—eg numbers potentially eligible, examined for eligibility, confirmed eligible, included in the study, completing follow-up, and analysed   ***Initial sample size calculation determined a minimum of 80 participants***  ***A cohort of 86 participants with CKD was recruited from the renal clinic***  ***All 86 participants were included in the study for analysis*** |
|  |  | (b) Give reasons for non-participation at each stage  ***NA*** |
|  |  | (c) Consider use of a flow diagram ***NA*** |
| Descriptive data | 14* | 1. Give characteristics of study participants (eg demographic, clinical, social) and information on exposures and potential confounders   ***Characteristics of Study Participants:***  ***Study involved 86 participants with CKD recruited from a renal clinic***  ***Participants were adults with renal pathologies, elevated serum creatinine, and blood urea levels***  ***Exposures and Potential Confounders:***  ***Exposures included thyroid hormone levels (TSH, T3, T4) and renal disease severity markers (creatinine, urea, PTH)***  ***Potential confounders were not explicitly mentioned in the provided sources.*** |
|  |  | (b) Indicate the number of participants with missing data for each variable of interest ***NA*** |
|  |  | (c) *Cohort study*—Summarise follow-up time (eg, average and total amount) ***The study was conducted from September to December 2023 at King Fahad Specialist Hospital in Tabuk City, Saudi Arabia*** |
| Outcome data | 15* | *Cohort study*—Report numbers of outcome events or summary measures over time  ***NA*** |
|  |  | *Case-control study—*Report numbers in each exposure category, or summary measures of exposure ***The study did not focus on a case-control design or report numbers in exposure categories.*** |
|  |  | *Cross-sectional study—*Report numbers of outcome events or summary measures  ***The study involved a cohort of 86 participants with CKD but did not report specific numbers of outcome events over time***  ***The study highlighted a generally weak correlation between thyroid hormones and indicators of renal disease severity, with Pearson correlation coefficients between -0.319 and 0.815***  ***Regression analyses showed weak correlations between serum creatinine levels and thyroid-stimulating hormone (TSH) and free thyroxine (T4) levels*** |
| Main results | 16 | 1. Give unadjusted estimates and, if applicable, confounder-adjusted estimates and their precision (eg, 95% confidence interval). Make clear which confounders were adjusted for and why they were included   ***The study found weak correlations between thyroid hormones and renal disease severity indicators, with Pearson correlation coefficients ranging from -0.319 to 0.815*** |
|  |  | 1. Report category boundaries when continuous variables were categorized   ***The study did not categorize continuous variables but analyzed them in their continuous form*** |
|  |  | (*c*) If relevant, consider translating estimates of relative risk into absolute risk for a meaningful time  Period  ***Absolute risk can be calculated by multiplying the relative risk by the baseline risk of the outcome over a specific time period*** |

Continued on next page

| Other analyses | 17 | Report other analyses done—eg analyses of subgroups and interactions, and sensitivity analyses ***NA*** |
| --- | --- | --- |
| **Discussion** |  |  |
| Key results | 18 | Summarise key results with reference to study objectives   - ***Weak correlation between thyroid hormones and renal disease severity markers was observed, with no significant association between creatinine and thyroid hormones*** - ***Strong positive correlation between parathyroid hormone and serum creatinine was noted, emphasizing the importance of considering PTH in managing CKD complications*** - ***Significant relationship between CKD, indicated by creatinine and urea levels, and parathyroid hormone was found, aligning with known pathophysiology*** - ***Tenuous associations between serum creatinine levels and thyroid-stimulating hormone (TSH), free thyroxine (T4), and triiodothyronine (T3) were observed*** - ***The study provided valuable insights into the intricate relationships among thyroid hormones, renal markers, and parathyroid hormone in individuals with CKD, highlighting the importance of monitoring thyroid function in these patients***. |
| Limitations | 19 | Discuss limitations of the study, taking into account sources of potential bias or imprecision. Discuss both direction and magnitude of any potential bias   - ***Small sample size of 86 participants may limit generalizability and statistical power*** - ***Lack of longitudinal evaluation may hinder understanding of long-term relationships between thyroid hormones and renal markers*** - ***Exclusion of individuals undergoing dialysis and those with systemic diseases could introduce selection bias*** - ***Potential confounding variables not accounted for in the analysis may affect the results*** - ***The study design being cross-sectional limits the ability to establish causality between thyroid hormones and renal disease severity*** |
| Interpretation | 20 | Give a cautious overall interpretation of results considering objectives, limitations, multiplicity of  analyses, results from similar studies, and other relevant evidence   - ***Weak correlation between thyroid hormones and renal disease severity markers suggests limited clinical implications*** - ***Strong positive correlation between parathyroid hormone and serum creatinine underscores the importance of considering PTH in managing CKD complications*** - ***Tenuous associations between serum creatinine levels and thyroid hormones indicate subtle relationships that may not be clinically significant.*** - ***Small sample size and lack of longitudinal evaluation limit the generalizability and causal inference of the findings.*** - ***Exclusion of certain patient groups and potential confounding variables may introduce bias and affect the robustness of the results.*** |
| Generalisability | 21 | Discuss the generalisability (external validity) of the study results   - ***Small sample size of 86 participants may limit the generalisability of the findings*** - ***Exclusion of individuals undergoing dialysis and those with systemic diseases could impact the external validity of the results*** - ***Lack of longitudinal evaluation may restrict the applicability of the findings to long-term outcomes*** - ***Potential bias from unaccounted confounding variables may affect the generalisability of the study results*** |
| **Other information** | | |
| Funding | 22 | Give the source of funding and the role of the funders for the present study and, if applicable, for the original study on which the present article is based   - **Researchers would like to thank the Deanship of Scientific Research at Qassim University for funding the publication of this project.** |

*Give information separately for cases and controls in case-control studies and, if applicable, for exposed and unexposed groups in cohort and cross-sectional studies.

**Note:** An Explanation and Elaboration article discusses each checklist item and gives methodological background and published examples of transparent reporting. The STROBE checklist is best used in conjunction with this article (freely available on the Web sites of PLoS Medicine at [http://www.plosmedicine.org/,](http://www.plosmedicine.org/) Annals of Internal Medicine at [http://www.annals.org/,](http://www.annals.org/) and Epidemiology at [http://www.epidem.com/).](http://www.epidem.com/)) Information on the STROBE Initiative is available at [www.strobe-statement.org.](http://www.strobe-statement.org/)
